# Estimating the Cost of the Design, Production, and Dissemination of Social Media Videos for Social and Behavioral Change: Evidence From Merci Mon Héros in Niger and Côte d'Ivoire

**DOI:** 10.3389/fpubh.2021.761840

**Published:** 2021-11-15

**Authors:** Michel Tchuenche, Nicole Bellows, Erin Portillo, Zamilatou H. Labati, Denise B. Adou, Jacqueline Hammond, Martha Silva, Lori Bollinger

**Affiliations:** ^1^Avenir Health, Glastonbury, CT, United States; ^2^Johns Hopkins Center for Communication Programs, Baltimore, MD, United States; ^3^Save the Children International, Niamey, Niger; ^4^Johns Hopkins Center for Communication Programs, Abidjan, Côte d'Ivoire; ^5^School of Public Health and Tropical Medicine, Tulane University, New Orleans, LA, United States

**Keywords:** reproductive health, family planning, social media, videos, social and behavior change, unit cost

## Abstract

Merci Mon Héros (MMH) is a youth-led multi-media campaign in Francophone West Africa seeking to improve reproductive health and family planning outcomes using radio, television, social media, and community events. One component to this project is the development of a series of youth-driven videos created to encourage both youth and adults to break taboos by talking to each other about reproductive health and family planning. A costing study was conducted to capture costs associated with the design, production, and dissemination of 11 MMH videos (in French) on social media in Côte d'Ivoire and Niger. The total costs to design, produce and disseminate 11 of the campaign videos for MMH in both Côte d'Ivoire and Niger were $44,981. Unit costs were calculated using three different denominators, resulting in average unit costs of $0.16 per reach, $1.29 per engagement, and $4.27 per video view. These findings can be useful for future studies of SBC interventions using social media for framing the analysis and selecting the appropriate metrics for the denominator, as well as for budgeting and planning SBC programs using social media.

## Introduction

Francophone West Africa continues to experience challenges in reproductive health and family planning (RH/FP), including high levels of maternal and child mortality, high unmet need for FP, and high fertility rates (1, 2). Adolescent pregnancy contributes to these poor health indicators by perpetuating intergenerational cycles of poverty and poor health (3). An increase in modern contraceptive prevalence rate (mCPR) among women in West Africa could help improve these health measures, however, uptake of FP has been low in this region, particularly among adolescents (4, 5). There are several factors driving the high fertility rate and low levels of FP use in the region, including poor access to FP services, negative attitudes toward FP, and religious and social norms surrounding fertility and early marriage (6–8). Due to cultural norms and taboos around RH, adolescents in sub-Saharan Africa typically do not have access to RH information via open dialogue with their parents, elders, or partners, which can improve knowledge and influence attitudes and behaviors (9).

A literature review of SBC strategies for improving RH/FP outcomes among youth ranging from adolescence to early adulthood in West Africa found evidence on the use and impact of traditional mass media interventions (e.g., radio and television), but limited evidence on interventions using social media platforms (10). Social media as a tool to convey (i.e., obtaining, sharing, or exchanging) information to the public can help inform or motivate health-related behavior change and/or influence health decision making as well as allow targeted messaging with hard-to-reach groups (11).

To fill this research gap, the United States Agency for International Development (USAID) is working in West Africa through the Breakthrough RESEARCH project to evaluate a mass multi-media campaign called Merci Mon Héros (MMH), which means “thank you, my hero” in French, being conducted through its sister project, USAID-funded Breakthrough ACTION. The MMH campaign includes radio, television, social media, and community events. One important component of MMH was the development of a youth-led campaign, beginning with a youth design challenge (YDC) at the Francophone SBC Summit in Abidjan, Côte d'Ivoire in February 2019. From this challenge evolved the “Merci Mon Héros” campaign wherein youth thank their parents, other family members, friends, teachers, and partners for communicating with them about RH and family planning (FP), and helping or supporting them when they had difficulty accessing RH/FP information or services (12). To help develop the campaign, the YDC winning team participated in a video production and dissemination-specific hands-on training on filming the videos (in French) with smartphones, interviewing, and post-production. Videos are not the MMH campaign's sole social media content, also included screenshots with key video quotes or messages as static visuals posted on social media to help increase the likelihood that key messages, themes, or ideas are captured. However, these components were not included in this analysis as the focus of the study was solely to capture costs associated with the design, production, and dissemination of the MMH videos on social media.

During the timeframe of the analysis, 11 MMH videos were disseminated via social media (e.g., Facebook, Twitter, YouTube, Instagram) and via radio, television, and community-based events in nine countries (Burkina Faso, Côte d'Ivoire, Togo, Niger, Democratic Republic of Congo, Guinea, Mali, Benin, and Senegal). The videos consisted of individuals recounting their experiences when someone in their life shared information about reproductive health issues and how this information helped them better understand their health and options. This study examines the cost of designing and disseminating the MMH videos via social media, with a focus on the videos' dissemination in Niger and Côte d'Ivoire. These two countries were selected for analysis because: (1) The YDC that sparked the original MMH campaign idea was held in Côte d'Ivoire, and (2) Niger has the highest total fertility rate in the world and high levels of adolescent pregnancy (13, 14). The results from this study will help fill important knowledge gaps on the costs of digital SBC interventions for health to improve coordinated investments in SBC for family planning and reproductive health (15).

The primary purpose of this study was to estimate the total design, implementation, and unit costs associated with the MMH videos. Many SBC costing studies neglect estimating the design costs of SBC interventions, and even less is known regarding design costs associated with RH/FP SBC programming with and for youth (15). Thus, these results will address this gap and help assist program planners considering similar approaches in the region.

A second objective was to examine the unit costs of MMH in Niger and Côte d'Ivoire. There are several potential program unit costs to examine with digital media, where the denominators of the unit costs (i.e., SBC campaign “reach”) are currently being discussed and conceptualized in the literature (16, 17). Unit costs are critical for budgeting and planning and defining unit costs has important implications for comparing costs across interventions (18–20). The different unit costs, based on different measures of reach, that can be used for SBC interventions delivered through social media are explored here to contribute to this new area of research.

## Materials and Methods

### Protocol and Data Collection Tool Development

The study protocol and data collection instruments were developed by Breakthrough RESEARCH and led by Avenir Health with support from MMH key country stakeholders. The data collection questionnaire was developed based on the SBC costing guidelines (21). Before data collection commenced, a series of web-based consultations were conducted with the Breakthrough ACTION team in Niger and Côte d'Ivoire, designed to gather their inputs and to evaluate whether the study instruments captured all relevant data. Feedback was documented and the data collection tool was subsequently revised and finalized.

### Approach and Cost Components

This costing study primarily took an economic costing approach, focusing on the resources needed to replicate the development of videos delivering SBC messaging through social media platforms in a similar setting. Included in the costs are those associated with in-kind contributions, such as donated personnel time and meeting space, which were valued at the expected costs based on information on the value of the donation provided by Breakthrough ACTION country teams. Broader societal costs were not included, such as the costs associated with the time for the intended audience to view the videos or the cost of internet access for users of social media.

Table 1 details the cost components included in this study from the time of conceptualization in late 2018 to dissemination through October 2020. Design costs included those associated with the YDC, training and production. The first set of costs includes personnel costs associated with preparing for and attending the February 2019 Abidjan SBC Summit, where the YDC committee selected the winning team, who were later invited to be co-creators of the resulting MMH campaign. Costs associated with the YDC include personnel and consultant time, travel, conference fees, room and catering fees, and stipends for 18 youth consultants/trainees who attended the summit. Since the attendees to the summit were not there exclusively to participate in the YDC, only a portion of the total conference costs were attributed to the campaign design, based on interviews with key staff.

**Table 1:** Cost components included in MMH video costing analysis.

**Cost category**	**Included components**	**Time frames**
Start-up and Youth Design Challenge	• Personnel time in preparation of the YDC (including donated time) • Personnel time at the YDC (including donated time) • Travel to the YDC, per diems, lodging • Conference and workshop costs (e.g., rooms, catering) • Stipends for youth	November 2018–February 2019
Training and production	• Personnel time (including donated time) • In-kind contributions • Travel and transport, per diems, lodging • Training venue and catering • Equipment	June 2019 and February 2020
Dissemination	• MMH launch cost • Personnel costs (youth consultants, influencers) • Social media advertising	November 2019–October 2020
Overhead	• Breakthrough ACTION overhead, including personnel not directly engaged in SBC activities (e.g., finance, human resources)	November 2019–October 2020

Subsequently, there was a workshop to strengthen capacity of the winning youth consultants, plus nearly a dozen additional young activists, journalists, and other young people working in the RH space in Francophone African countries for 2 weeks in June and July 2019. In addition, there was a 1-week refresher training on video production and dissemination for a group of paid youth consultants in February 2020. The costs associated with training and production were primarily the personnel time associated with staff and consultant trainers, travel, lodging and catering, car hires, and equipment. Equipment costs were relatively low as the videos were filmed using smart phones and open-source software; the costs of four smartphones purchased by Breakthrough ACTION and used for MMH were included in the analysis.

The first MMH video was posted on Facebook in November 2019. Dissemination costs associated with the core MMH videos include personnel costs associated with social media influencers/bloggers paid to disseminate and promote the videos, social media paid advertising, and costs associated with the MMH launch in each country (e.g., equipment rental, services). While the dissemination of the videos and production of new videos continues at the time of this manuscript, the data collection for the dissemination phase reported here includes costs from November 2019 through October 2020 only.

Finally, a portion of the overhead costs associated with Breakthrough ACTION's oversight of the production and dissemination of the videos were included. These include rent and support personnel (e.g., finance, human resources) costs to support activities for the purpose of planning, coordinating, and managing youths' technical work on MMH video production.

### Data Collection

Because of the ongoing COVID-19 pandemic, the costing team worked remotely with Breakthrough ACTION headquarters and the Côte d'Ivoire and Niger teams to collect the data using the data collection instrument. Despite the communication challenges of remote data collection, in-country stakeholders' engagements commenced via a series of calls and web consultations in July 2020 to review the questionnaire and discuss the required data. The web consultations also familiarized the country teams with the data collection tool and the informed consent form. Verbal informed consent was obtained from key informants, which primarily included in-country SBC program and financial managers for Breakthrough ACTION.

Starting in September 2020, the team conducted extensive in-country stakeholder engagements via a series of phone calls, emails, and web consultations to review the data provided. Where data were incomplete, the research team requested clarifications from MMH stakeholders, both at headquarters as well as in-country.

Data on the reach of the MMH videos were captured via social listening reports conducted by Breakthrough RESEARCH in partnership with M&C Saatchi Intelligence (22). Their final campaign summary report captured social media engagement with MMH videos from November 1, 2019 through October 20, 2020 and included the number of persons reached, video views, likes, retweets, shares, and other forms of engagement by country, based on user IP addresses.

### Analysis

Cost data obtained from the data collection instruments were entered into a Microsoft Excel workbook for analysis. The total design and production costs were captured and allocated to the two study countries using the proportion of the level of effort the production team (youth consultants) and the in-country Breakthrough ACTION team spent on the conceptualization, production, and review of the videos during the analysis period. Because of the potential recall bias for respondents to accurately estimate their level of effort to support the different stages of design, production, and dissemination of MMH videos, a sensitivity analysis around this estimate was carried out by varying the level of effort in both direction by ± 5%, that is if the level of effort was estimated to be 10%, for example, the sensitivity analysis examines a range from 5 to 15%.

Costs allocated to the design and production of the videos include costs associated with the YDC and trainings for creating the videos, including personnel costs for these activities. To apportion the design and production costs for Côte d'Ivoire and Niger from the total costs of the overall MMH program, the percentage of the overall design and production costs allocated to each of the two countries was based on the relative reach to these countries, based on the social listening dissemination report, with 18% in Côte d'Ivoire and 6% in Niger.

The apportioned design and production costs were added to the total dissemination costs for each country. Dissemination costs include costs associated with the in-person campaign launch events, social media advertising, social influencers/bloggers, and program personnel for these activities. The launches were in-person but meant to drive interest in the broader campaign as well as the digital campaign. As such, only a portion of the launch costs were attributed to the dissemination of the videos, based on input from key staff. For costs listed in local currency, the average exchange rate for 2019/2020 of 552.47 FCFA = US$1 was used (23).

Unit costs were calculated using an ingredients-based costing approach, where all inputs were listed, their costs collected, and the contribution of these costs to the overall cost were quantified. For SBC disseminated *via* social media, measuring cost per exposure is particularly interesting due to the nature of social media platforms, which measures exposure differently from other SBC approaches, such as mass media and interpersonal communication. Three different program output denominators that measure exposure were used to calculate unit costs for Côte d'Ivoire and Niger:

- **Reach**—the number of people who saw MMH posts at least once; meaning any content from the MMH page entered their screen, including when people scroll past the post quickly.
- **Engagements**—the number of times people engaged with MMH posts through reactions, comments, shares, retweets, mentions, and likes.
- **Views**—the number of times the eleven videos were viewed for at least 30 s, where each video was at least 2 min long (max four and half minutes).

To calculate the number for each denominator for Côte d'Ivoire and Niger, the breakdown in Facebook users who have engaged with MMH content by location was used to best approximate country-specific denominators.

## Results

The total cost to design, produce and disseminate the 11 core videos for MMH in both Côte d'Ivoire and Niger from November 2018 through October 2020 was $44,981, as shown in Table 2. Among the categories shown, the highest proportion of costs across both countries are for overhead and consultants (each 35% for a total of 70%). The distribution of costs was mostly consistent between Côte d'Ivoire and Niger. Costs can also be disaggregated into two categories: (1) design and production and (2) implementation and dissemination (see Table 3). For both Côte d'Ivoire and Niger, ~60% of the costs went into design and production and 40% went into dissemination.

**Table 2:** Total design, production, and dissemination costs.

	**Côte d'Ivoire**		**Niger**	
**Cost Category**	**USD**	**Percentage**	**USD**	**Percentage**
Personnel	3,105	10	1,072	8
Consultants	10,206	33	5,422	39
Travel and transport	903	3	213	2
Social media advertising	2,633	8	827	6
Campaign launch	899	3	1,079	8
Training (non-personnel)	2,511	8	549	4
Overhead	11,076	35	4,586	33
Total	31,592	100	16,707	100

**Table 3:** Costs, denominators, and unit costs (USD).

	**Côte d'Ivoire**	**Niger**
**Costs**		
Design and production	18,840	8,173
Implementation	12,394	5,575
Total	31,233	13,748
**Denominators**		
Reach	206,645	73,802
Engagements	26,074	9,312
Views	7,890	2,818
**Unit costs**		
Cost per reach	0.14	0.18
Cost per engagement	1.12	1.45
Cost per view	3.75	4.79

**Figure 1:**
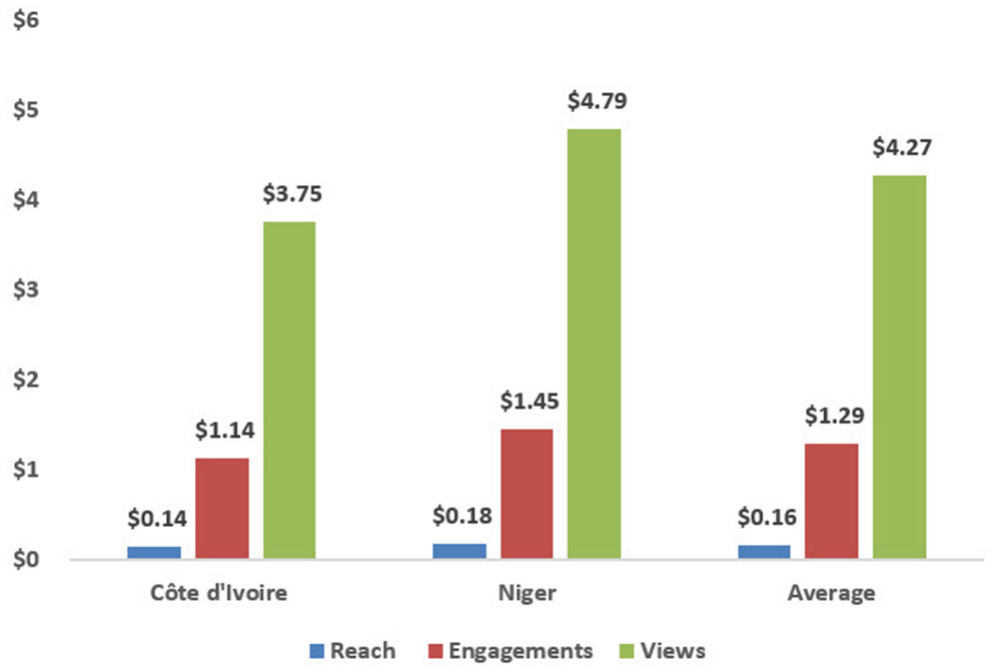
MMH video unit cost by exposure level.

Unit costs are presented using three different denominators in Table 3. The average unit cost per person reached via social media across both countries was $0.16 (Côte d'Ivoire $0.14 and Niger $0.18), while the average unit cost per engagement was $1.29 (Côte d'Ivoire $1.14 and Niger $1.45). Finally, the average unit cost per 30+ second view was $4.27 (Côte d'Ivoire $3.75 and Niger $4.79). Figure 1 further shows the unit costs for each country in graphical format.

Results of the sensitivity analysis around the respondent estimated level of effort by either a unilateral increase or decrease by 5% in the level of effort allocated to MMH videos are shown in Table 4. Based on the sensitivity analysis, the average unit cost ranged from $0.15 to $0.17 per reach, $1.23 to $1.36 per engagement, and $4.06 to $4.48 per view.

**Table 4:** Unit costs range of MMH videos.

**Denominator**	**Côte d'Ivoire**	**Niger**	**Average**
Reach	$ 0.14–0.15	$ 0.17–0.19	$ 0.15–0.17
Engagements	$ 1.08–1.19	$ 1.38–1.52	$ 1.23–1.36
Views	$ 3.57–3.94	$ 4.55–5.03	$ 4.06–4.48

**Figure 2:**
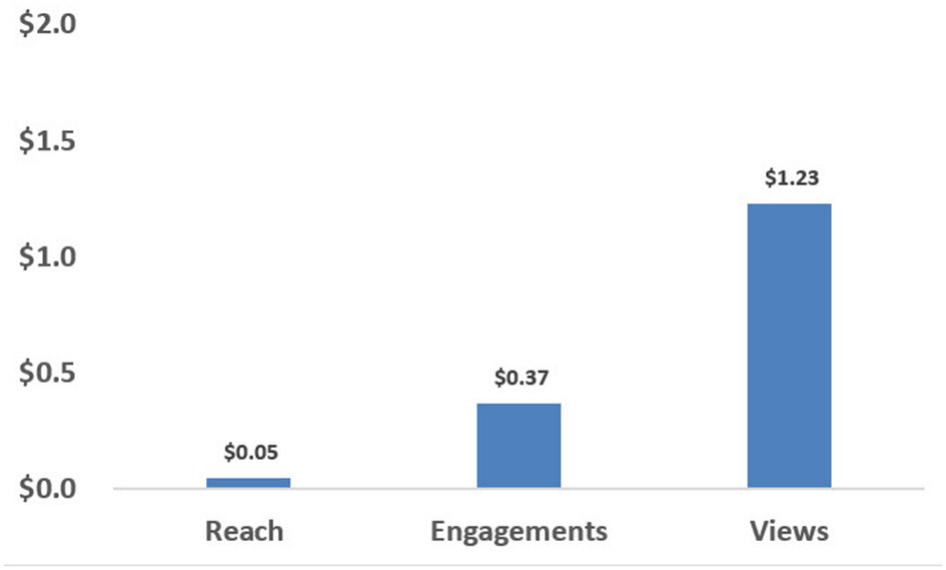
Regional design and production unit cost.

Since the total costs of the design and production of the MMH videos were captured for all the West African core countries, the aggregate unit costs associated with design and production can also be examined using data capturing outcomes across all of the countries where MMH was disseminated. This total unit design and production cost was $57,525. When divided by the total outcome variables, the unit costs for design and production were $0.05 per reach, $0.37 per engagement, and $1.23 per view (see Figure 2).

## Discussion

The use of social media for disseminating SBC messages has greatly increased in recent years, with many publications describing the uses, benefits, and limitations of social media for achieving public health objectives (16). Few studies, however, have examined the measurement of reach and/or costs when using social media. Researchers have considered different measures of social media and have acknowledged that there are a variety of outcome metrics available and a lack of agreement on which is the most appropriate for gauging the impact of a social media campaign (16, 17). In a literature review on SBC costs, no studies were identified that examined the unit costs associated with SBC campaigns using social media (15). As such, it is difficult to benchmark the unit cost findings from this research to other SBC campaigns on social media; however, it is useful to think about the results and their implications for SBC costing.

Approximately one third of the costs associated with MMH was spent on design and production and two thirds on dissemination in Côte d'Ivoire and Niger. The $57,525 total spent on design and production for the region was apportioned to Niger and Côte d'Ivoire based on country specific program reach. As such, it is important to consider the total costs for design/production when considering the findings for budgeting and planning purposes, as country-specific costs can contain efficiencies from having a regional program or approach. As dissemination continues over time and as more videos are produced leveraging the ever-deepening knowledge from the original training sessions, the design and production costs per output will decrease as these initial fixed costs are spread over more persons engaging with and viewing the videos. One of the novel approaches undertaken by MMH is the collaboration with youth in the design, production, and dissemination of the MMH videos. While the counterfactual of what it would cost to design and produce MMH without the involvement with youth was not assessed in this analysis, any additional costs associated with youth-led SBC should be weighed against the economic benefits of building capacity in the next generation of SBC professionals in the region. These benefits are not captured in this analysis but have important implications for potential societal returns on investment.

The unit costs for Côte d'Ivoire and Niger are very similar, with slightly higher unit costs in Niger due to the lower reach of the MMH videos in Niger compared to Côte d'Ivoire. While the design and production costs were allocated based on country reach, dissemination costs had fewer people reached, engagements, and views to be spread over. This is apparent in the higher proportion of total costs in the campaign launch costs in Niger vs. Côte d'Ivoire. The lower reach of SBC via social media in Niger is likely a function of more limited internet connectivity in Niger relative to Côte d'Ivoire and other countries in the region, where only 2% of the population is on Facebook in Niger compared to 18% in Côte d'Ivoire (24).

Finally, the unit cost results using the three different denominators highlight the different potential approaches for assessing social media costs. The cost per reach denominator measures the cost to have the MMH videos appear on an individual's screen. By “reaching” an individual, one may scroll past the video without absorbing the content; however, by having the message on their social media feed, they have the opportunity to further engage with the content. The “engagement” denominator indicates an individual is noticing the content based on likes, shares, and comments. Finally, the “view” denominator examines how many times the video was viewed for longer than 30 s.

The unit cost results indicate that the MMH videos were inexpensive to reach people with an average of $0.16 per person reached. This is comparable to the median unit costs for traditional SBC mass media campaigns per person exposed ($0.17 for television, $0.26 for radio, $0.25 billboards/flyers) (15). In the world of social media, however, reaching someone's screen is not likely sufficient for a person to absorb and process the campaign's message, as social media feeds are abundant with other content simultaneously competing for one's attention.

The second measure is the $1.29 average cost per “engagement,” which reflects the cost to get people to acknowledge the MMH campaign by either sharing, liking, or commenting. While “engagement” can capture a range of intensity from a simple “like” to a more participatory conversation through comments, this aggregated measure appears to be a more accurate gauge of campaign exposure. A remaining question is whether individuals who are engaging with the MMH content without fully watching the videos are still receiving the basic messages of MMH around destigmatizing and normalizing conversations about RH. Thus, future SBC impact analysis could investigate to what extent “engagement” is associated with improved knowledge, beliefs, and attitudes that the videos are addressing.

Finally, the $4.27 per video view is the most expensive outcome; the unit costs are comparable to SBC unit costs for group interpersonal communication (15). The higher cost per video view, compared to reach and engagements, is driven by the lower number of “views” for at least 30 s. In the West Africa context, this may be due to the fact that viewers usually have out-of-pocket costs on internet credit to access social media video content. Most individuals need to pre-purchase internet bundles for viewing videos and other media and watching videos can quickly drain internet credit. As such, after getting a sense of the primary message of the campaign without watching them for long, viewers may be stopping the videos early to conserve internet credit or because the videos are not engaging their attention.

### Limitations

While efforts were made to produce a comprehensive cost analysis of SBC, several limitations to this analysis should be noted. First, the cost data on the MMH videos were aggregated with costs from the broader MMH campaign and estimates of level of effort were used to isolate the costs of the MMH videos' design, production, and dissemination for the two focal countries of Côte d'Ivoire and Niger. However, these estimates may lack precision and have the potential for error. Contributing to potential imprecision is the possibility of recall bias. Staff supporting the MMH activities do not work solely on this program; respondents needed to accurately estimate their level of effort to support the design, production, and dissemination of MMH videos. Because no time-motion study was carried out to assess how staff and youth consultants spent their time on the MMH videos, a sensitivity analysis was used to explore this imprecision and found the 5% variability did not have substantial impact on the unit costs.

Second, this study is novel in that no prior research was identified quantifying the unit costs associated with SBC via social media. As such, it is impossible to benchmark these results against the costs of other SBC campaigns via social media studies. The costs presented here are unique to this particular social media campaign, and a different set of videos with a different subject matter, length, or use of professionals would likely vary greatly from those presented in this analysis. While the unit costs described here can be considered against the backdrop of other SBC interventions such as “per person exposed” for mass media or “per person participating” for IPC, these unit costs are not directly comparable since they have different denominators. More research is needed on the effectiveness, costs, and cost-effectiveness of SBC using social media to appropriately benchmark MMH to similar programs. Only when several studies emerge on the costs of SBC interventions disseminated via social media, will there be enough information to properly gauge how the costs of SBC vary when using social media vs. more traditional forms of SBC dissemination.

A third limitation is that this analysis is narrowed to include only the MMH videos where there is denominator data to generate unit costs. However, MMH is not exclusively a digital campaign nor are the videos the campaign's sole digital content, but rather has complementary mass media and community-based activities. While neither the cost nor the reach of these non-digital activities is included in the analysis, there are potential areas where synergies between these program components are not being captured. For example, if the videos are screened at a community event, the number of people who viewed the videos during that event are not being captured in the analysis.

## Conclusion

This study examining the cost of designing, production and disseminating the core MMH videos via social media in Niger and Côte d'Ivoire is the first to examine the unit costs of an SBC social media campaign and can be used by future research to compare findings from similar approaches, despite the above limitations. The variety of available denominators makes this a particularly interesting area for new research; thus, this study suggests that the selection of denominator is critical in determining unit cost estimates. Looking forward, SBC programmers should investigate which denominator(s) (e.g., reach, engagement, views) provide the best measure to capture based on the program objectives. Additionally, future research that pairs effectiveness and cost studies on SBC via social media are essential to better understand the utility of this channel for promoting positive behavior change.

## Data Availability Statement

The original contributions presented in the study are included in the article/supplementary material, further inquiries can be directed to the corresponding author.

## Author Contributions

NB and MT conceptualized and designed the study, conducted the literature search, and wrote the original manuscript. MT, NB, EP, ZL, AD, MS, and LB had roles in the questionnaire design. MT conducted data collection virtually, data analysis, and data interpretation. EP, ZL, AD, and JH critically reviewed the data collected. All authors contributed to the writing and improvement of the data interpretation of the manuscript, read, and approved the final version.

## Funding

Breakthrough RESEARCH is made possible by the generous support of the American people through the United States Agency for International Development (USAID) under the terms of cooperative agreement no. AID-OAA-A-17-00018.

## Author Disclaimer

The contents of this document are the sole responsibility of the Breakthrough RESEARCH and Population Council and do not necessarily reflect the views of USAID or the United States Government.

## Conflict of Interest

The authors declare that the research was conducted in the absence of any commercial or financial relationships that could be construed as a potential conflict of interest.

## Publisher's Note

All claims expressed in this article are solely those of the authors and do not necessarily represent those of their affiliated organizations, or those of the publisher, the editors and the reviewers. Any product that may be evaluated in this article, or claim that may be made by its manufacturer, is not guaranteed or endorsed by the publisher.
